# Increased risk of osteoporosis in patients with cognitive impairment: a systematic review and meta-analysis

**DOI:** 10.1186/s12877-023-04548-z

**Published:** 2023-12-04

**Authors:** Chengxin Xie, Chenglong Wang, Hua Luo

**Affiliations:** 1 Department of Orthopedics, Taizhou Hospital of Zhejiang Province Affiliated to Wenzhou Medical University, Linhai, 317099 China; 2 Key Laboratory of Endocrine Glucose & Lipids Metabolism and Brain Aging, Department of Endocrinology, Ministry of Education, Shandong Provincial Hospital Affiliated to Shandong First Medical University, Jinan, 250021 China; 3 Department of Orthopedics, Taizhou Central Hospital (Taizhou University Hospital), Taizhou, 318001 China

**Keywords:** Osteoporosis, Bone loss, Cognitive impairment, Alzheimer’s disease, Dementia, Meta-analysis

## Abstract

**Background:**

Both osteoporosis and cognitive impairment affect overall health in elderly individuals. This study aimed to investigate the association between cognitive impairment and the risk of osteoporosis.

**Methods:**

PubMed, Web of Science, and the Cochrane Library were searched for studies on the association between osteoporosis and cognitive impairment from their inception until August 2023. The random-effects model was used to calculate the pooled risk ratio (RR) of osteoporosis in patients with cognitive impairment. Subgroup analysis was used to detect the sources of heterogeneity. Sensitivity analysis was used to test the robustness of the pooled results. Funnel plots, Egger’s test, and Begg’s test were used to test publication bias.

**Results:**

Ten studies involving 9,872 patients were included in this meta-analysis. The pooled results showed that patients with cognitive impairment had an increased risk of osteoporosis (RR = 1.56, 95% confidence interval [CI]: 1.30–1.87, *p* < 0.001). Subgroup analysis showed that patients with Alzheimer’s disease (AD) are at 1.7-fold risk of osteoporosis compared with the control group (RR = 1.70, 95% CI: 1.23–2.37, *p* = 0.001), and sex, cognitive classification, study region, study design, and study quality might be the sources of heterogeneity. Sensitivity analysis showed robustness of the pooled results. No significant publication bias was found (Begg’s test, *p* = 0.474; Egger’s test, p = 0.065).

**Conclusion:**

Current evidence suggests that patients with cognitive impairment are at increased risk of osteoporosis, especially patients with AD.

**Supplementary Information:**

The online version contains supplementary material available at 10.1186/s12877-023-04548-z.

## Background

Osteoporosis is the most common metabolic bone disorder and is characterized by a reduction in bone mineral density (BMD), deterioration of bone microarchitecture, and accumulation of marrow fat, which subsequently leads to increased bone fragility and fracture susceptibility [1, 2]. Osteoporosis and osteoporotic fracture pose great medical, public health, and economic burdens worldwide [1, 3, 4]. In China, the prevalence of osteoporosis among females ranged from 37.1% among those aged 60 to 69 years to more than 67.5% among females older than 80 years [4]. During the aging process, increased adipogenesis of bone marrow mesenchymal stem cells at the expense of osteogenesis is a key factor leading to bone fat imbalance and ultimately osteoporosis [5].

Neurodegenerative disorders, such as Alzheimer’s disease (AD), Parkinson’s disease, and amyotrophic lateral sclerosis, are characterized by the progressive degeneration of neurons in the central nervous system. Numerous studies have extensively investigated the contribution of neurodegenerative disorders in various peripheral disorders [6–10]. Moreover, these neurodegenerative disorders can have adverse effects on bone health. Over the past decade, several epidemiological studies have highlighted the potential connection between neurodegenerative disorders and the development of osteoporosis [11–23]. These studies have provided evidence of reduced BMD and increased fracture risk in individuals with neurodegenerative conditions [11, 14–22].

AD, the most common neurodegenerative disorder, is mainly characterized by a progressive decline in memory and cognitive function and accounts for 75% of all dementia cases [12, 24]. Globally, dementia affects approximately 1.8%, 5.1%, 15.1%, and 35.7% of individuals in their 60s, 70s, 80 and 90 s, respectively [25]. The worldwide prevalence of dementia is estimated at 50 million people, and by 2050, it is projected to double in Europe and triple worldwide [24]. With the increasing global life expectancy, the prevalence of osteoporosis and dementia is expected to continue rising.

The causal relationship between BMD and cognitive function remains unclear. Clinically, osteoporosis shares many risk factors with AD, including older age, post menopause, obesity, type 2 diabetes, hypomagnesemia, smoking, alcohol abuse, chemotherapy, lack of physical activity, deficiency of vitamin D and K, and APOE4 genotype, among others [2, 13, 26–29]. However, these risk factors cannot fully explain this comorbidity. Zhao et al. [30] investigated individuals with osteoporosis and specifically explored the prevalence of cognitive impairment in this population. Their meta-analysis revealed that elderly individuals with osteoporosis have a significantly increased risk of cognitive impairment (OR = 2.01, *p* < 0.01) [30].

Recently, we found the study by Liu et al. [31] to be of great interest as it provided insights into the mechanism of AD-induced bone loss. Their study identified the role of AD brain-derived extracellular vesicles (EVs) as a promoter of osteoporosis by transferring miR-483-5p [31]. Therefore, we speculate that AD individuals are also at an increased risk of osteoporosis. However, to date, there have been no meta-analyses investigating whether the prevalence of osteoporosis increases among individuals with cognitive impairments. Therefore, we conducted this meta-analysis to confirm this association.

## Methods

This study followed a methodology guided by the Preferred Reporting Items for Systematic Reviews and Meta-Analyses (PRISMA) 2020 statement [32]. We publicly registered a formal protocol for the current study in the PROSPERO database (CRD42023448414). Two authors independently screened the studies, extracted the data, and assessed their quality. Any disagreement was resolved by consensus with a third author.

### Eligibility criteria

Inclusion criteria: clinical studies included cohort studies, case‒control studies, and cross-sectional studies; the exposed group was patients with any type of cognitive impairment; the control group was patients with normal cognitive function; all participants’ clinical data included diagnostic information on osteoporosis; only studies published in English were considered; and patients of any age were considered.

The exclusion criteria were as follows: duplicate literature; literature without full text; erroneous or incomplete data; studies with an unclear diagnosis of osteoporosis or cognitive impairment; patients with pathological fractures or bone tumors; and study sample size < 300.

### Search strategy

We searched PubMed, Web of Science, and the Cochrane Library for studies from inception until August 2023. The search terms and Boolean operators used were as follows (in PubMed): (Alzheimer’s disease [Title/Abstract] OR dementia [Title/Abstract] OR cognitive impairment [Title/Abstract]) AND (osteoporosis [Title/Abstract] OR bone loss [Title/Abstract] OR bone density [Title/Abstract]). The search strategies were adjusted accordingly to suit each database. Furthermore, we also manually checked the reference lists of related articles to identify additional eligible studies.

### Study selection

EndNote X9 software was used to manage the literature. We imported literature entries into the software and then removed the duplicate literature. Ineligible studies were excluded by an initial screening of the title and abstract. Finally, we determined eligible studies that met our eligibility criteria based on a full-text review of the remaining studies.

### Data extraction

The following data were extracted into a standardized Microsoft Excel spreadsheet: first author, year of publication, country of origin, study design, sample size, age, sex, tool of cognitive assessment, site of osteoporosis assessment, and number of patients with bone loss (osteoporosis and/or osteopenia).

### Quality evaluation

The methodological quality (MQ) of the selected studies was evaluated using the Newcastle‒Ottawa Scale (NOS) [33]. In this tool, eight items were covered among the selection of cohorts/cases (four items), comparability of cohorts/cases (one item), and assessments of outcomes (three items), with a total score ranging from 0 to 9. The NOS has been widely used to evaluate MQ in cohort and case‒control studies. The Appraisal Tool for Cross-Sectional Studies (AXIS) is a popular MQ tool for cross-sectional studies [34]. However, there was no evidence to suggest that the AXIS confers advantages over the NOS for cross-sectional studies [35]. Hence, we uniformly used the NOS for MQ evaluation.

### Outcomes

The primary outcome was the incidence of osteoporosis (T Score ≤ − 2.5). In addition, we collected data on osteopenia (− 2.5 < T Score ≤ − 1.0) and bone loss (T Score ≤ − 1.0). According to the World Health Organization criteria, in the clinic, a T Score > − 1.0, − 2.5 < T Score ≤ − 1.0, or T Score ≤ − 2.5 was referred to as normal, osteopenia, or osteoporosis, respectively [36].

### Statistical analysis

The meta-analysis was conducted in the following steps. First, study heterogeneity was measured using the *I*^*2*^ statistic, and an *I*^*2*^ value ≥ 50% indicated significant and substantial heterogeneity. Given the significant heterogeneity, a meta-analysis was performed using a random-effects model. Second, the risk ratio (RR) and 95% confidence interval (CI) were determined to calculate the effect sizes. Third, subgroup analysis and sensitivity analysis were used to explore sources of heterogeneity. Finally, a funnel plot was used to detect publication bias [37]. In addition, Begg’s and Egger’s tests were used to quantitatively test for publication bias. All statistical analyses were performed using Stata 12.0. Statistical significance was defined as two-tailed *p* < 0.05.

## Results

### Literature search

**Fig. 1:**
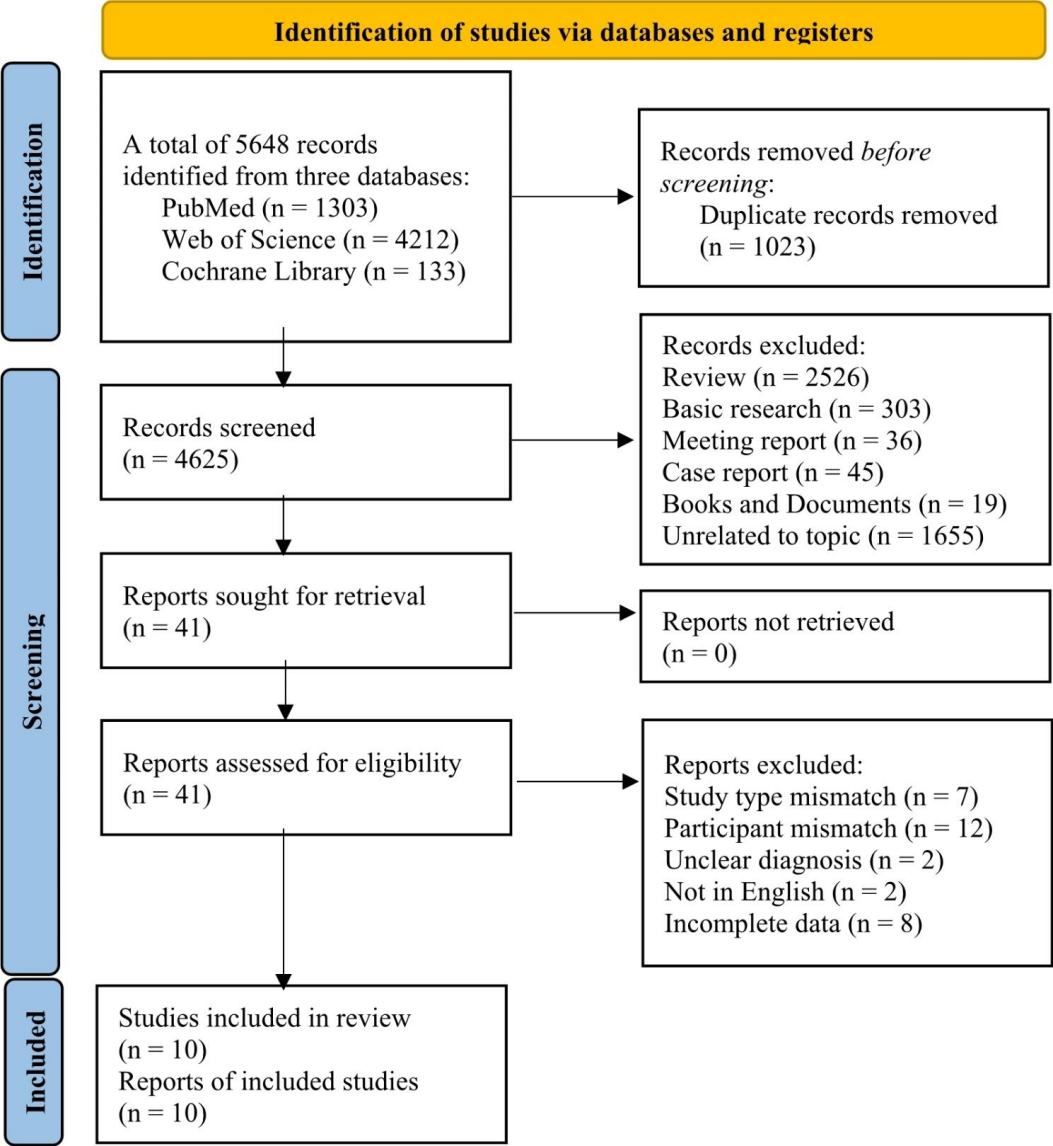
PRISMA flow chart of study screening

A total of 5648 studies were retrieved, including 1303 studies in PubMed, 4212 studies in Web of Science, and 133 studies in the Cochrane Library. After 1023 duplicate studies were removed, we read the titles and abstracts of the remaining 4625 studies and excluded studies that did not meet our eligibility criteria. We then reviewed the full text of the 41 studies and further excluded ineligible studies. Finally, 10 studies that fulfilled the predefined eligibility criteria were included in this meta-analysis. Following the PRISMA 2020 flow diagram, we showed the study screening process in Fig. 1.

### Characteristics of the included studies

The basic characteristics of these 10 studies are summarized in Table 1. These studies were from seven countries and were published between 2016 and 2022. Of these, three were cohort studies, three were case‒control studies, and four were cross-sectional studies. The sample size for each study was ≥ 300. The average age of all participants was > 62 years. Six studies used the Mini-Mental State Examination (MMSE) to assess cognitive function. Seven studies recorded the location details of the BMD assessment.

**Table 1:** Characteristics of the included studies

Study	Country	Study design	Sample size (n)	Age (years)		Female [n (%)]	Cognitive assessment	Location of BMD
			CI/Control					
Li 2016 [14]	China	Case‒control	345 1380	≥ 65	78.9 ± 6.1 78.9 ± 6.1	139(40.3) 722(52.3)	MMSE ≤ 26 MHIS ≤ 4	Lumbar spine
Liu 2016 [15]	China	Cohort	257 1545	≥ 60	70.9 ± 4.9 65.3 ± 4.6	141 (54.9) 708 (45.8)	MMSE < 24	Vertebral and femoral neck
Kang 2018 [16]	Korea	Cross-sectional	170 480	> 50	65.3 ± 7.2 62.1 ± 8.1	138(81.2) 320(66.7)	MMSE < 24	Lumbar spine and femur
Mughal 2019 [17]	Australia	Cohort	226 276	≥ 65		178(78.8) 204(73.9)	AMTS < 7	
Basgoz 2020 [18]	Turkey	Cross-sectional	93 270	≥ 65	78.4 ± 5.1 78.7 ± 6.0	58(62.4) 169(62.6)	MMSE < 24	Lumbar spine, total hip, and femoral neck
Ebrahimpur 2020 [19]	Iran	Cohort	677 831	≥ 60	71.11 ± 7.26 67.58 ± 5.18	437(64.55) 297(35.74)	Mini-Cog CFT ≤ 12	Lumbar spine, total hip, and femoral neck
Rasu 2020 [20]	USA	Cross-sectional	526 1649	≥ 65		400(76.0) 1279(77.6)	Dementia diagnosis	
Kumar 2021 [21]	Pakistan	Case‒control	150 150		65 ± 10 64 ± 12	75(50) 70(47)	MMSE	
Lin 2021 [22]	China	Cross-sectional	175 360	≥ 65	- 71.3 ± 5.6	95(54.3) 138(38.3)	MMSE ≤ 26	Lumbar spine and femoral neck
Jiang 2022 [23]	China	Case‒control	117 195		67.72 ± 5.02 67.91 ± 5.79	80(68.4) 127(65.1)	AD diagnosis	Lumbar spine and total hip

*MMSE* Mini-Mental State Examination, *AMTS* Abbreviated Mental Test Score, *MHIS* Modified Hachinski ischemia scale, *CFT* Categorical verbal fluency test

### Quality evaluation

The results of MQ evaluation based on the NOS tool are shown in Table 2. Five studies were considered to be of high quality (☆ ≥ 7), while the remaining five were of moderate quality (4 ≤ ☆ ≤ 6).

**Table 2:** Quality assessment of studies using the Newcastle Ottawa scale

Study	Selection	Comparability	Outcome	Total score
Li 2016 [14]	☆☆☆	☆	☆☆	6
Liu 2016 [15]	☆☆☆☆	☆	☆☆☆	8
Kang 2018 [16]	☆☆☆	☆☆	☆☆	7
Mughal 2019 [17]	☆☆☆	☆	☆☆☆	7
Basgoz 2020 [18]	☆☆☆	☆☆	☆	6
Ebrahimpur 2020 [19]	☆☆☆☆	☆☆	☆☆☆	9
Rasu 2020 [20]	☆☆	☆	☆☆	5
Kumar 2021 [21]	☆☆	☆☆	☆☆	6
Lin 2021 [22]	☆☆☆	☆☆	☆☆☆	8
Jiang 2022 [23]	☆☆☆	☆☆	☆	6

High quality: ☆ ≥ 7; moderate quality: 4 ≤ ☆ ≤ 6; poor quality: ☆ ≤ 3

### Outcomes

**Fig. 2:**
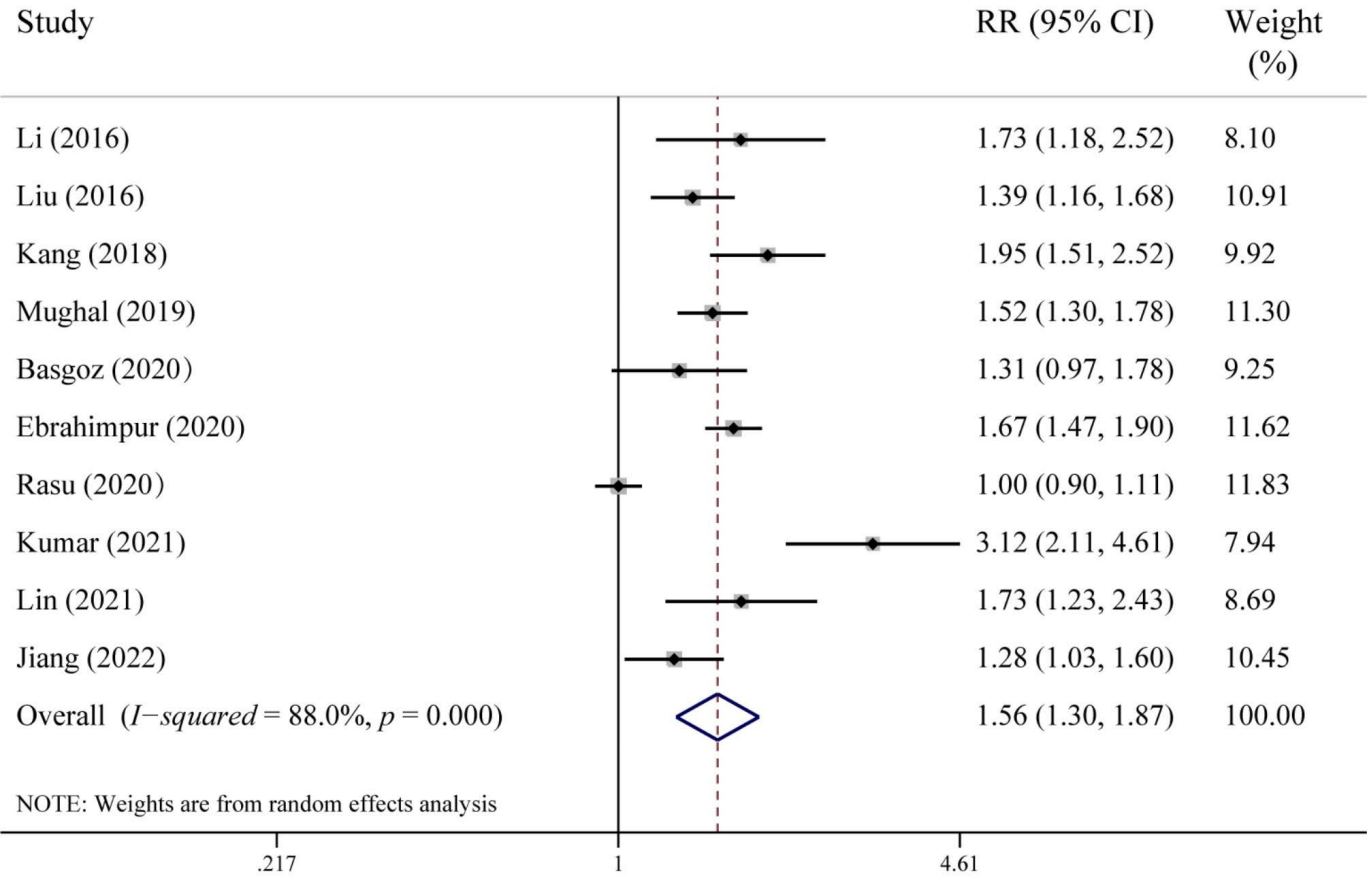
Forest plot of the association between cognition impairment and risk of osteoporosis

Ten studies with a total sample size of 9,872 reported data on the incidence of osteoporosis. The overall prevalence of osteoporosis in patients with cognitive impairment was 42.8%, while in the control group, it was 27.6%. As shown in Fig. 2, there was significant statistical heterogeneity among the trials (*I*^*2*^ = 88.0%, *p* < 0.001); thus, we used a random-effects model to perform the meta-analysis. The pooled results showed that patients with cognitive impairment were at higher risk of osteoporosis than the control group (RR = 1.56, 95% CI: 1.30–1.87, *p* < 0.001).

**Fig. 3:**
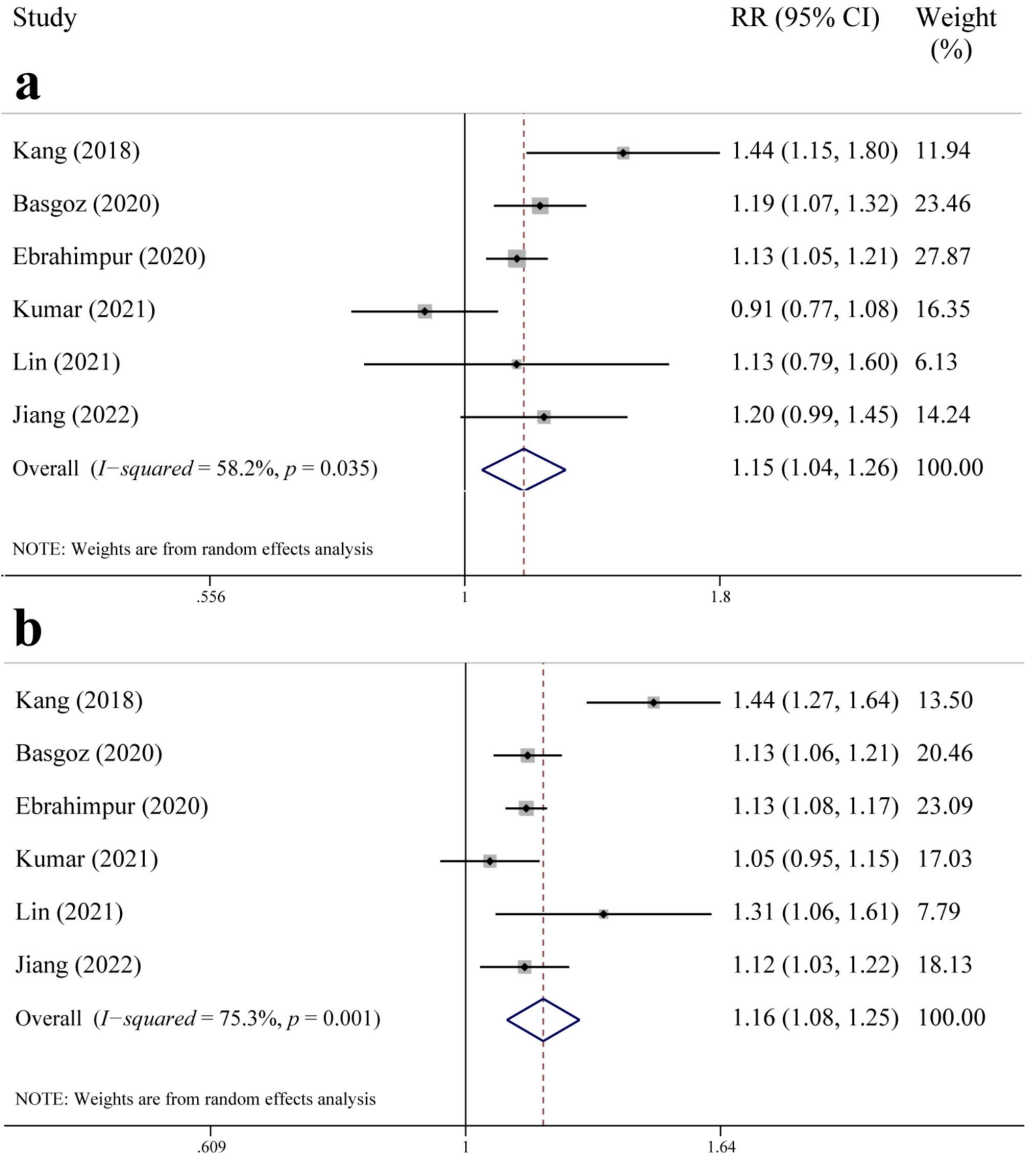
Forest plot of the association between cognition impairment and risk of (**a**) osteopenia and (**b**) bone loss

Six studies provided data on the incidence of osteopenia and/or bone loss. As shown in Fig. 3a, there was significant statistical heterogeneity among the studies on osteopenia (*I*^*2*^ = 58.2%, *p* = 0.035); the pooled results from a random-effects model showed that patients with cognitive impairment had a higher risk of osteopenia (RR = 1.15, 95% CI: 1.04–1.26, *p* = 0.006). As shown in Fig. 3b, based on significant statistical heterogeneity among the studies on bone loss (*I*^*2*^ = 75.3%, *p* = 0.001), we used a random-effects model to pool the results and found that patients with cognitive impairment had a higher risk of bone loss (RR = 1.16, 95% CI: 1.08–1.25, *p* < 0.001).

### Subgroup analysis

Subgroup analysis of the risk of osteoporosis was performed according to different factors, such as age, sex, cognitive classification, region, sample size, study design, and study quality. We found that *I*^*2*^ values significantly changed in the subgroups of sex, cognitive classification, study region, study design, and study quality, which indicated that these factors might be the sources of heterogeneity. The results of the subgroup analysis are shown in Table 3 and Supplementary Fig. 1 to 7. Patients with AD had a high risk of osteoporosis (RR = 1.70, 95% CI: 1.23–2.37, *p* = 0.001).

**Table 3:** Subgroup analysis of the association between cognition impairment and risk of osteoporosis

Factors	Subgroup	Studies	Heterogeneity		Pooled results	
		(n)	*I* ^*2*^	*p* value	RR (95% CI)	*p* value
Age (years)	≥ 65	5	86.3%	< 0.001	1.40 (1.08, 1.81)	0.011
	≥ 60	2	60.1%	0.114	1.55 (1.30, 1.85)	< 0.001
	> 50	1			1.95 (1.51, 2.52)	< 0.001
Sex	Female	2	50.6%	0.155	1.30 (1.10, 1.54)	0.002
	Male	2	17.4%	0.271	1.22 (0.85, 1.76)	0.282
Cognition	Impairment	3	25.7%	0.260	1.66 (1.48, 1.85)	< 0.001
	MCI	1			1.48 (0.97, 2.28)	0.073
	Dementia	3	85.2%	0.001	1.34 (0.91, 1.98)	0.144
	AD	4	82.6%	0.001	1.70 (1.23, 2.37)	0.001
Region	East Asia	5	48.4%	0.101	1.56 (1.32, 1.83)	< 0.001
	Western Asia	2	52.5%	0.147	1.54 (1.23, 1.93)	< 0.001
	South Asia	1			3.12 (2.11, 4.61)	< 0.001
	Other	2	94.8%	< 0.001	1.23 (0.81, 1.86)	0.327
Sample size (n)	< 1000	6	75.3%	0.001	1.68 (1.36, 2.08)	< 0.001
	≥ 1000	4	92.9%	< 0.001	1.40 (1.03, 1.89)	0.032
Study design	Case‒control	3	87.8%	< 0.001	1.87 (1.10, 3.21)	0.022
	Cohort	3	24.6%	0.265	1.55 (1.40, 1.72)	< 0.001
	Cross-sectional	4	89.9%	< 0.001	1.43 (0.99, 2.07)	0.055
Study quality	Moderate	5	89.8%	< 0.001	1.51 (1.07, 2.12)	0.018
	High	5	26.1%	0.247	1.61 (1.46, 1.77)	< 0.001

*MCI* Mild cognitive impairment, *AD* Alzheimer’s disease

### Sensitivity analysis

**Fig. 4:**
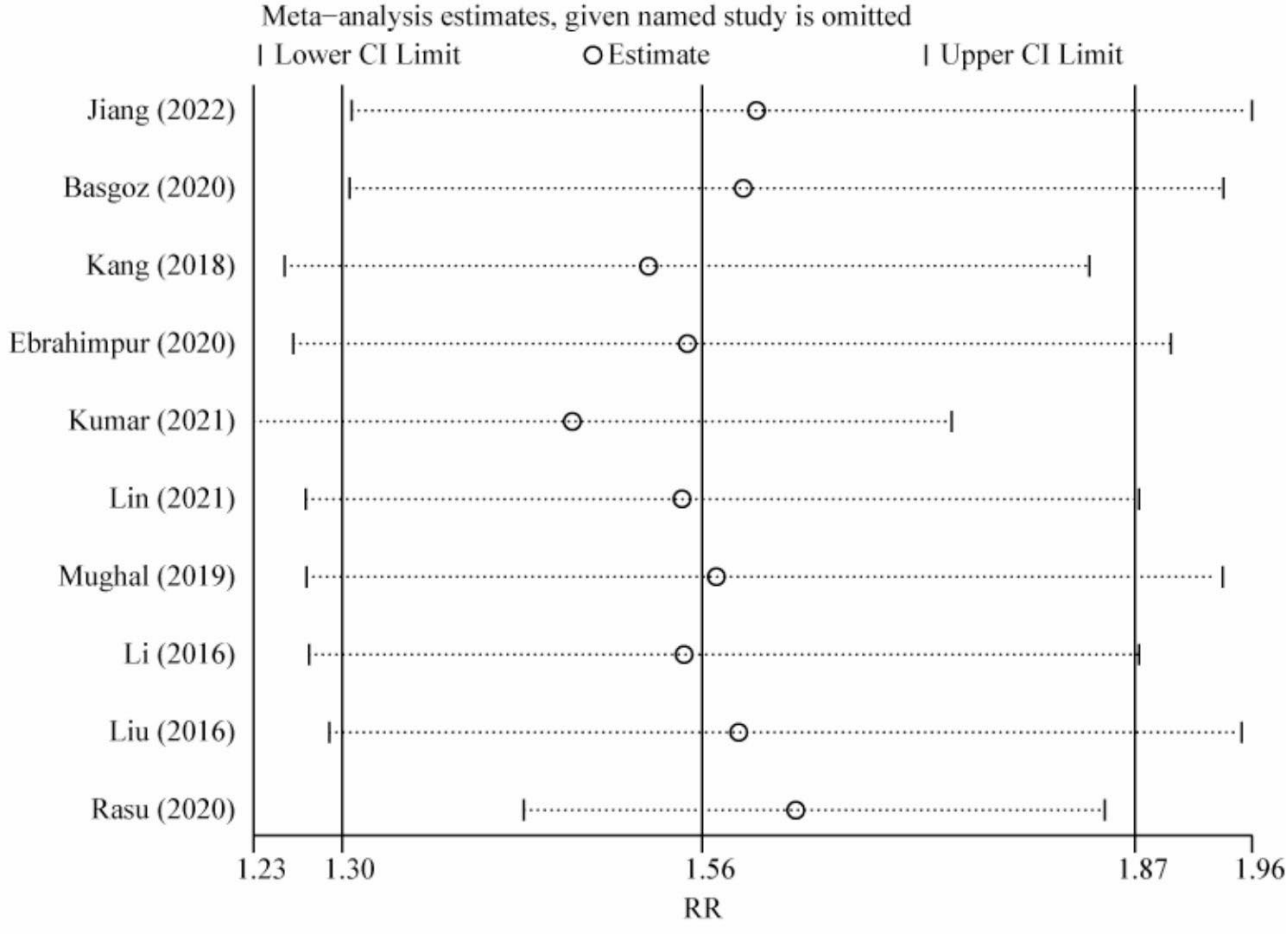
Sensitivity analysis of the association between cognition impairment and risk of osteoporosis

Given that significant heterogeneity existed (*I*^*2*^ = 88.0%, *p* < 0.001), a sensitivity analysis based on the leave-one-out strategy was performed to test the robustness of the pooled results of the risk of osteoporosis. As shown in Fig. 4, there was no significant change in RRs and 95% CIs each time a single study was removed from the pooled analysis. Thus, the pooled results of this meta-analysis were relatively robust.

### Publication bias

**Fig. 5:**
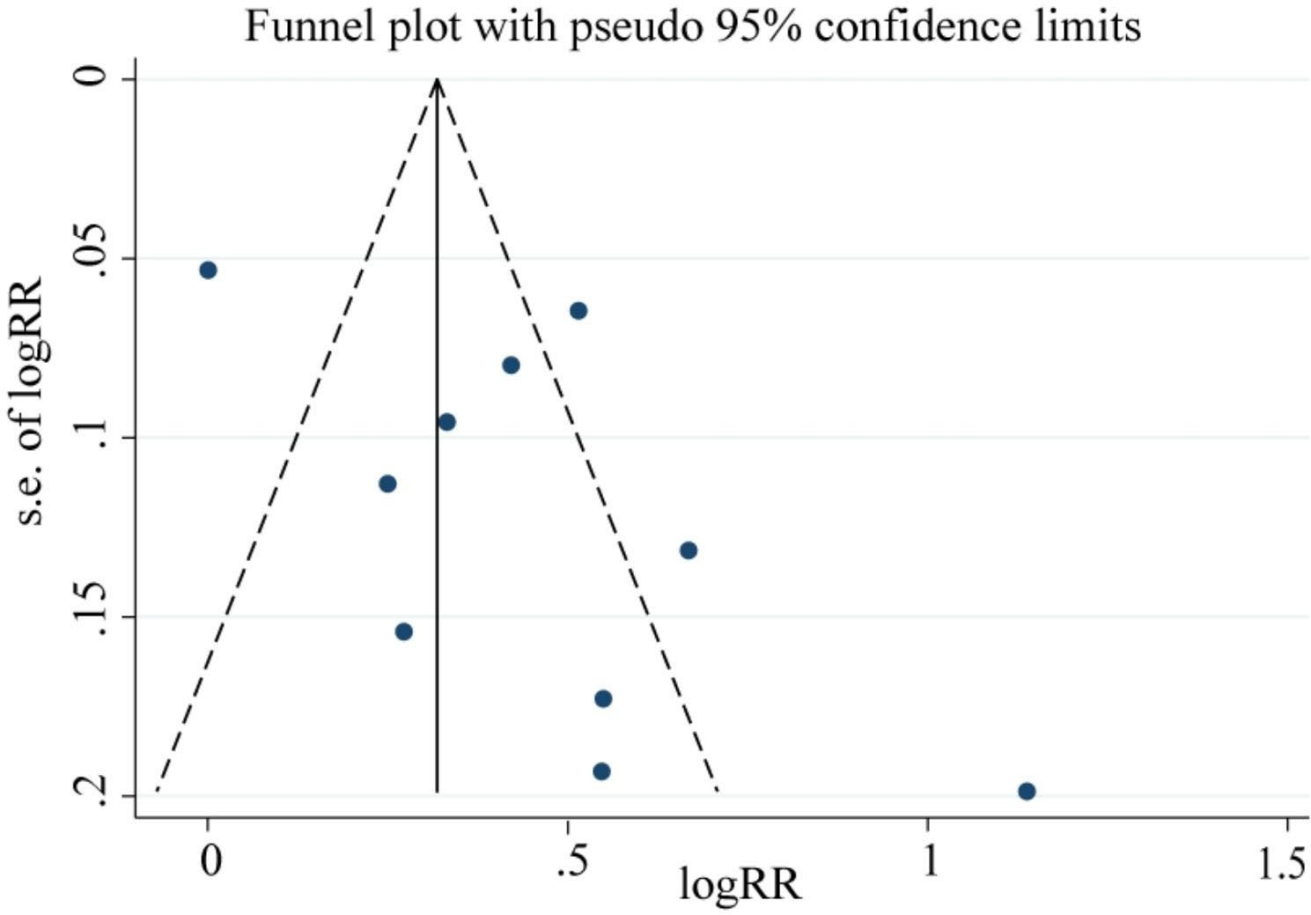
Funnel plot for detecting publication bias

Based on visual observations, we found that the funnel plot was asymmetric (Fig. 5), indicating that there might be a potential risk of publication bias. Further quantitative tests showed that there was no significant publication bias across studies (Begg’s test, *p* = 0.474, Supplementary Fig. 8; Egger’s test, p = 0.065, Supplementary Fig. 9).

## Discussion

To our knowledge, this is the first study to investigate the risk of osteoporosis in patients with cognitive impairment using a meta-analytical technique. In the current study, 9,872 patients from 10 eligible studies were included in the final analysis. The pooled results showed a significantly increased risk of osteoporosis in patients with cognitive impairment. In addition, we also found an increased risk of osteopenia in these patients. Subgroup analysis indicated that sex, cognitive classification, study region, study design and study quality may be the main sources of heterogeneity.

Osteoporosis and AD are two common geriatric syndromes, and skeletal fragility has been recognized as a comorbidity in AD [26, 38]. Although a two-sample Mendelian randomization study showed that there is no genetic causal relationship between osteoporosis and AD [39], recent clinical and preclinical studies have shown that these two conditions are closely related [12, 23, 30, 31, 40]. Kwon et al. [12] found a 1.49-fold higher prevalence of AD in osteoporosis individuals over 40 years old than in the control group after adjusting for age and sex, indicating that the presence of osteoporosis increases the potential for AD. Several possible mechanisms for the association between osteoporosis and cognitive impairment have been proposed [41, 42]. A novel mechanism in bone-brain communication may explain this clinical relevance. Osteocyte-derived EVs can be transported to the brain under physiological and pathological conditions and ameliorate cognitive impairment and pathogenesis in AD mice [23]. Unfortunately, EVs derived from senescent osteocytes lost their protective functions [23].

Interestingly, brain-derived EVs are also involved in the regulation of bone metabolism. Recently, Liu et al. [31] found that brain-derived EVs from AD mice crossed the blood‒brain barrier to reach the distal bone tissue, leading to a bone-fat imbalance and ultimately resulting in osteoporosis. AD is pathologically characterized by the appearance of amyloid-β (Aβ) plaques and neurofibrillary tangles. Aβ is also expressed in nonneural tissues, including osteoblasts and osteoclasts, which can directly impair osteoblast proliferation and increase osteoclast activation [43, 44]. AD patients commonly exhibit increased sympathetic tone and reduced parasympathetic activation [45], and impaired parasympathetic signaling leads to bone homeostatic imbalance [46]. These potential mechanisms support the findings of the current study, which suggest an increased risk of osteoporosis in AD patients (RR = 1.70, *p* = 0.001).

The subgroup analysis found lower heterogeneity in the cohort study group and the high MQ group, suggesting that more well-designed studies are needed to confirm our findings. We found no significant association between osteoporosis and cognitive impairment in the male subgroup. Since the average age of the included population was > 62 years, loss of estrogen protection might be the major cause of osteoporosis in postmenopausal women. In addition, our subgroup analysis showed that the RR of osteoporosis was 1.40, 1.55, and 1.95 in subgroups ≥ 65 years old, ≥ 60 years old, and > 50 years old, respectively. There are many possible confounders in elderly individuals, which may impact our results on the correlation between osteoporosis and cognitive impairment. Therefore, future research should collect more younger individuals, especially those between 40 and 60 years old. Importantly, among the ten included studies, eight were conducted in Asian countries. Subgroup analysis indicated significant differences in all subgroups of Asian countries (all *p* < 0.001). However, no significant difference was observed in the other subgroup (p = 0.327). These findings suggest that the association between osteoporosis and cognitive impairment may be influenced by ethnic differences.

Our study has several strengths. First, this is the first meta-analysis to support the high risk of osteoporosis in patients with cognitive impairment, especially AD patients. Second, each study included in this meta-analysis had moderate or high MQ, and its sample size was relatively large. Third, our subgroup analysis is multiperspective, and its results may provide novel ideas for future research design. In addition, the results of sensitivity analysis and publication bias testing confirm that our conclusions are robust. Importantly, our results are consistent with previous clinical and basic studies, which contribute to the great confidence of the findings. However, several limitations should be treated with considerable caution. First, the bias caused by different design of study can affect our conclusions. Second, there was considerable heterogeneity among the studies, warranting a careful interpretation of the findings. Third, we did not limit the criteria of cognitive impairment for the included studies. Fourth, all subjects in the current study were over 50 years old, and future research needs to reanalyze the difference in younger individuals. Finally, most of these included studies were conducted in Asian countries, which may weaken the applicability of our conclusions. Current evidence only supports the association between cognitive impairment and an increased incidence of osteoporosis within the Asian population. Although we performed subgroup analysis to clarify the state of the evidence, more well-designed large-scale studies are still needed to validate our findings in the future.

## Conclusions

In conclusion, our findings support that patients with cognitive impairment are at increased risk of osteoporosis, especially patients with AD. We encourage early treatment of neurodegenerative diseases to improve cognitive function, avoiding bone loss caused by brain aging. However, considering the high heterogeneity between the included studies, more meticulously designed studies are needed.

### Electronic supplementary material

Below is the link to the electronic supplementary material.

**Supplementary Material 1:** Subgroup analyses and publication bias test

## Data Availability

All data analysed during this study are available from the published article [8–17].

## List of abbreviations

AD: Alzheimer’s disease
BMD: Bone mineral density
RR: Risk ratio
CI: Confidence interval
PRISMA: Preferred reporting items for systematic reviews and meta-analyses
MQ: Methodological quality
NOS: Newcastle‒Ottawa Scale
AXIS: Appraisal Tool for Cross-Sectional Studies
MMSE: Mini-Mental State Examination
AMTS: Abbreviated Mental Test Score
MHIS: Modified Hachinski ischemia scale
CFT: Categorical verbal fluency test
EVs: extracellular vesicles
Aβ: amyloid-β
