# Supplementary material for: Increased risk of osteoporosis in patients with cognitive impairment: a systematic review and meta-analysis
Source: BMC Geriatr. 2023 Dec 4;23:797. doi: 10.1186/s12877-023-04548-z (PMC10694915; doi:10.1186/s12877-023-04548-z)
Supplement: Supplementary file 1 — Supplementary Material 1: Subgroup analyses and publication bias test [file 12877_2023_4548_MOESM1_ESM.docx]

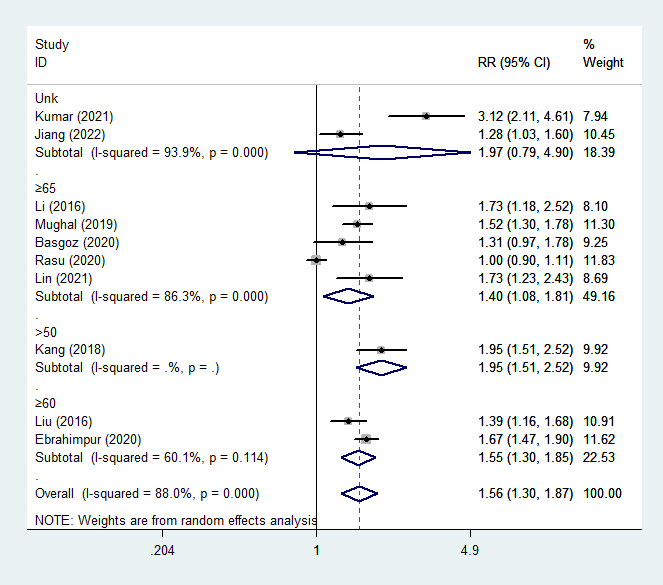


**Supplementary Fig 1.** Subgroup analysis of the osteoporosis risk by age.


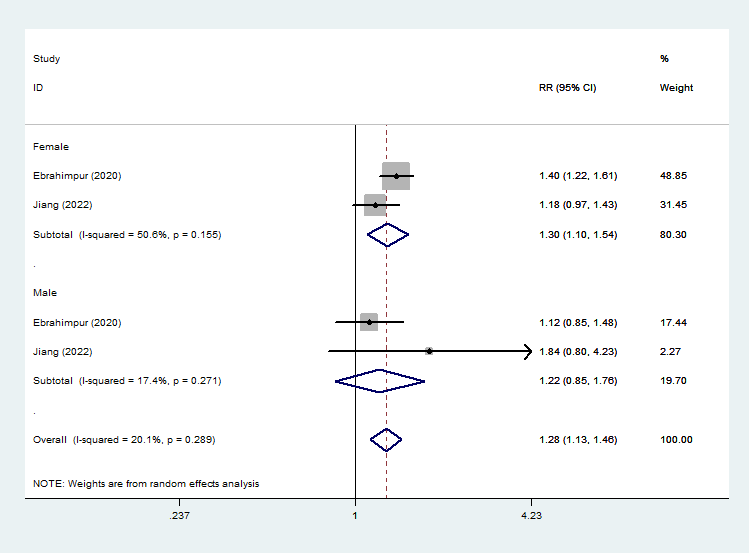


**Supplementary Fig 2.** Subgroup analysis of the osteoporosis risk by sex.


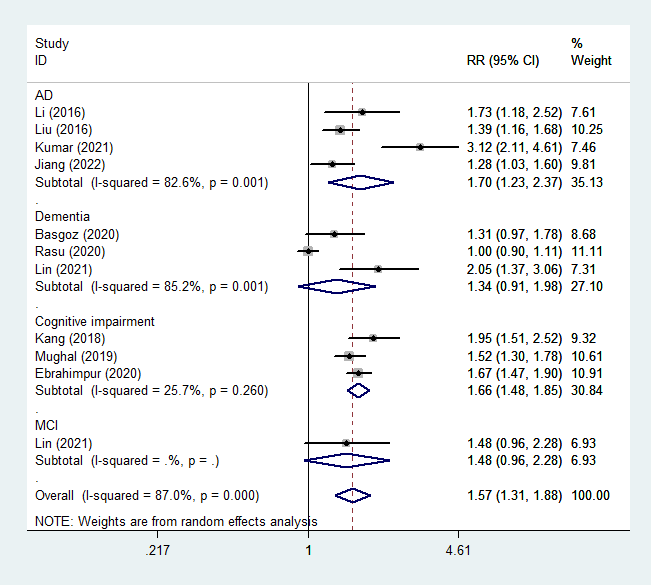


**Supplementary Fig 3.** Subgroup analysis of the osteoporosis risk by cognitive classification.


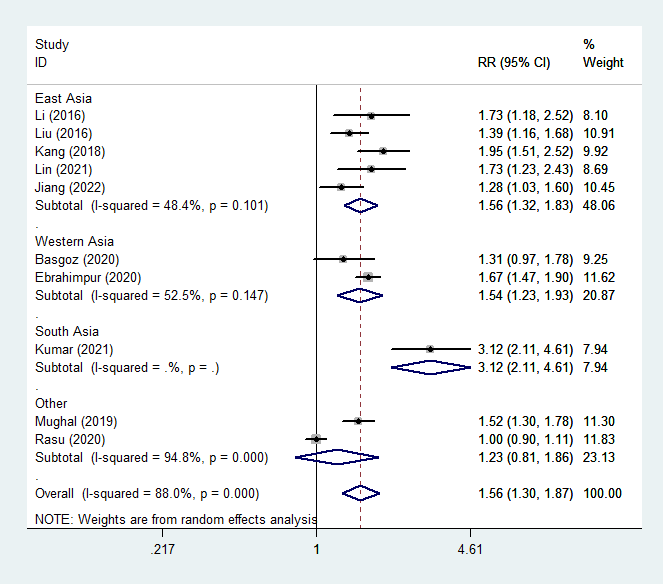


**Supplementary Fig 4.** Subgroup analysis of the osteoporosis risk by study region.


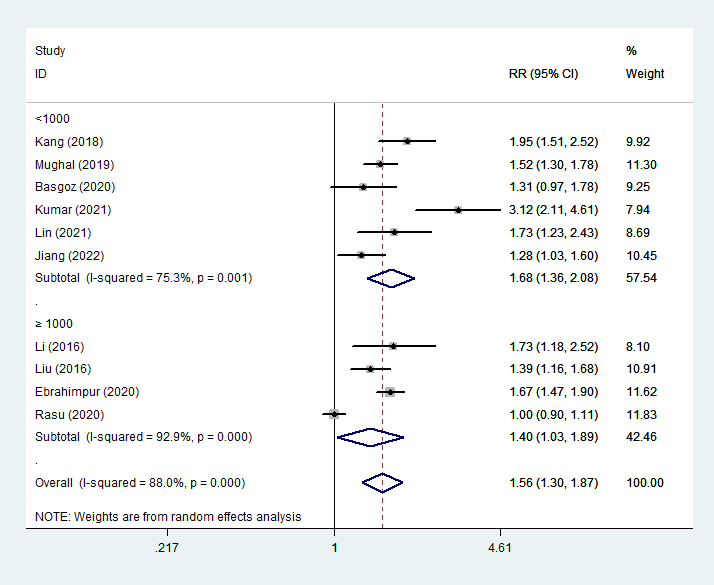


**Supplementary Fig 5.** Subgroup analysis of the osteoporosis risk by study size.


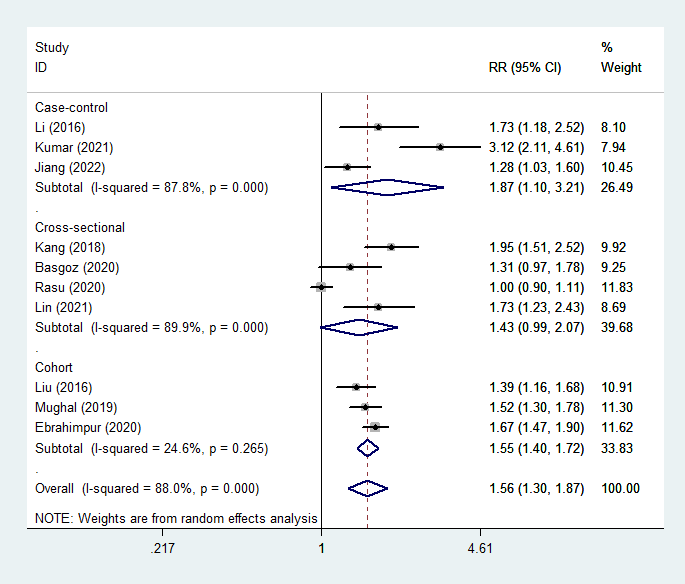


**Supplementary Fig 6.** Subgroup analysis of the osteoporosis risk by study design.


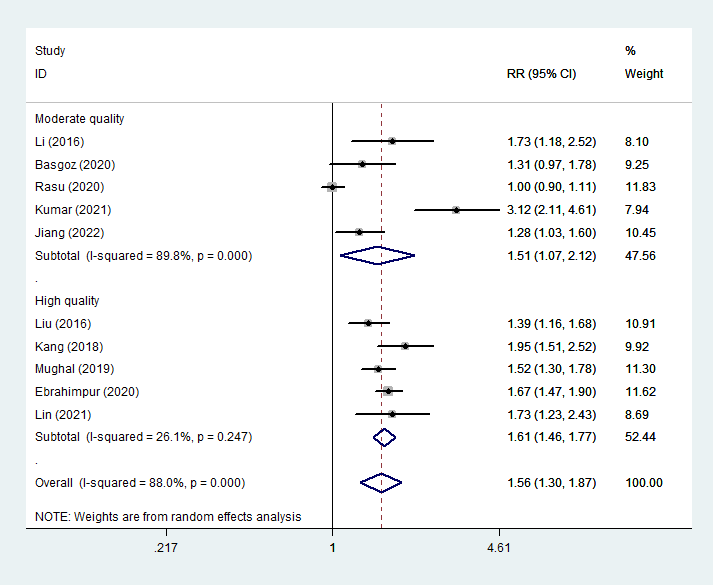


**Supplementary Fig 7.** Subgroup analysis of the osteoporosis risk by study quality.


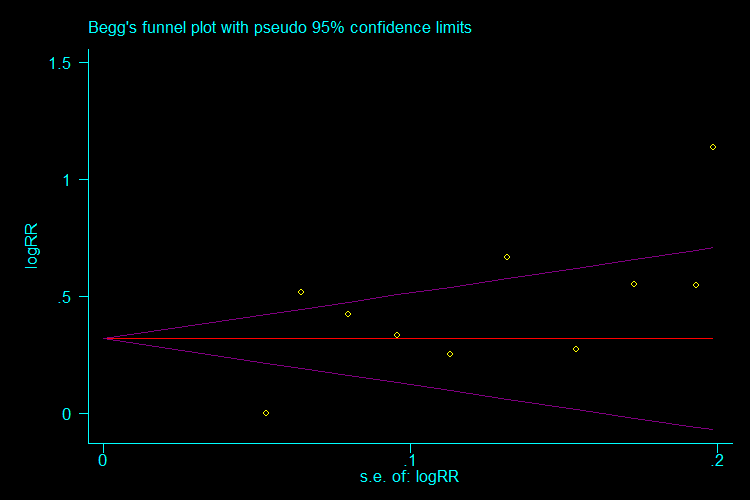


**Supplementary Fig 8.** Quantification of publication bias by Begg’s test.


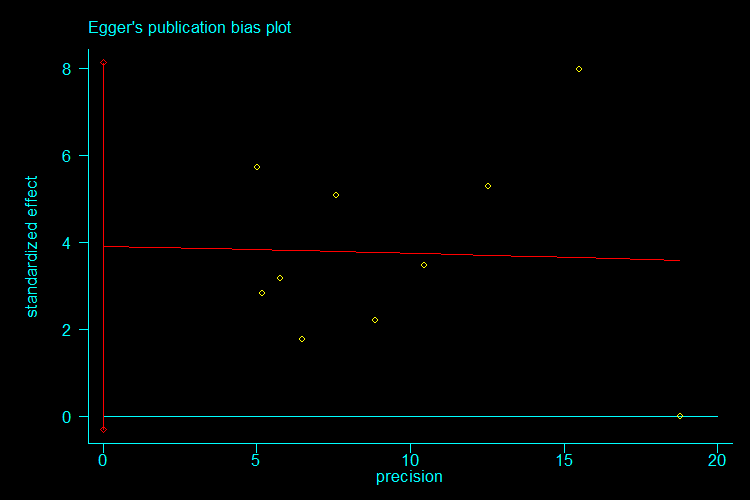


**Supplementary Fig 9.** Quantification of publication bias by Egger’s test.
